# Endoscopic debulking of a large colonic lipoma causing recurrent intussusception using endoscopic mucosotomy technique

**DOI:** 10.1055/a-2094-8435

**Published:** 2023-06-15

**Authors:** Jenson Phung, Morgan Freeman, Mohammad Bilal

**Affiliations:** 1 Department of Internal Medicine, University of Minnesota Medical Center, Minneapolis, Minnesota, United States; 2 Division of Gastroenterology, Hepatology, and Nutrition, University of Minnesota Medical Center, Minneapolis, Minnesota, United States; 3 Division of Gastroenterology and Hepatology, Minneapolis Veterans Affairs Medical Center, Minneapolis, Minnesota, United States

Colonic lipomas are usually incidental findings during colonoscopy. Lipomas ≥ 4 cm can cause obstruction and intussusception. Surgical resection is often recommended in these instances 1 2 . However, endoscopic resection of larger lipomas has been reported previously using PolyLoop-assisted unroofing technique, snare catheter unroofing/resection, and placement of endoclips prior to resection 3 . Here, we describe a novel technique for endoscopic debulking of a large colonic lipoma.

A 63-year-old man with a known large colonic lipoma causing recurrent intussusception presented with abdominal pain. He had previously declined surgery. Computed tomography scan of the abdomen demonstrated an 8-cm fat-containing mass in the ascending colon. Colonoscopy showed a large trilobed mass in the ascending colon with areas of ulceration, occluding the entire lumen of the colon ( Fig. 1 ). The patient refused surgery, and after multidisciplinary discussion, the decision was made to attempt endoscopic resection.

**Fig. 1:**
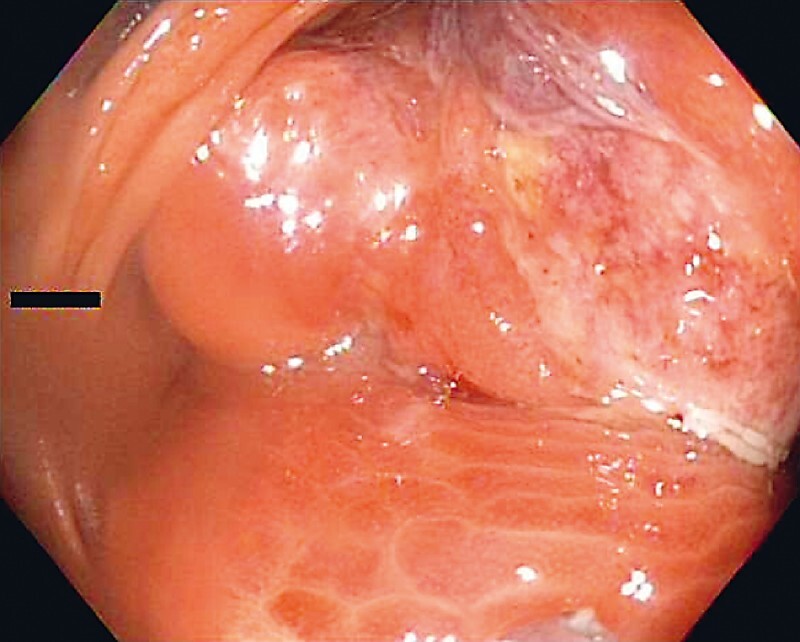
Large lipoma obstructing the lumen of the ascending colon.

A colonoscopy was performed, and attempts to place a PolyLoop (Olympus, Tokyo, Japan) around the mass were unsuccessful due to the size and smoothness of the mass. Therefore, the decision was made to use an endoscopic submucosal dissection knife to perform a mucosal incision on the surface of the lesion to expose the underlying fatty tissue ( Fig. 2 a ). The lipoma was eventually exposed, and the fatty tissue was resected piecemeal with a snare ( Fig. 2 b ). The lipoma was significantly debulked with resolution of the patient’s abdominal pain.

**Fig. 2:**
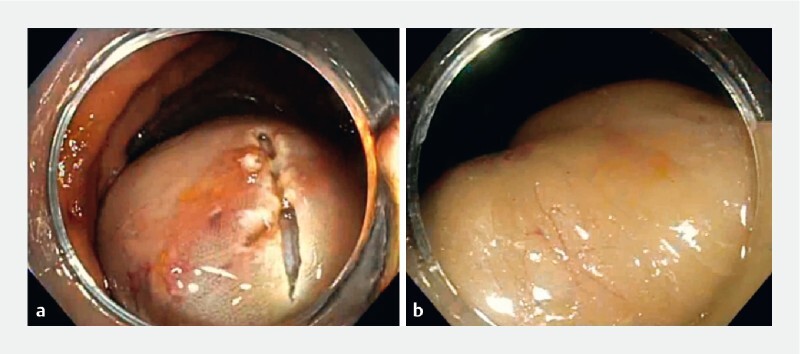
Endoscopic images. **a** The initial mucosal incision. **b** The exposed lipoma following mucosotomy.

Repeat colonoscopy was performed after 8 weeks, and the lesion was significantly smaller. Debulking was performed again using the same technique. As the size of the lesion was significantly reduced compared with the original lipoma, two PolyLoops could be successfully placed over the lesion, allowing sloughing of the remainder of the lesion.

Further colonoscopy after 6 months showed significant improvement in luminal narrowing ( Fig. 3 , Video 1 ). The patient had no recurrence of intussusception at the 6-month follow-up.

**Fig. 3:**
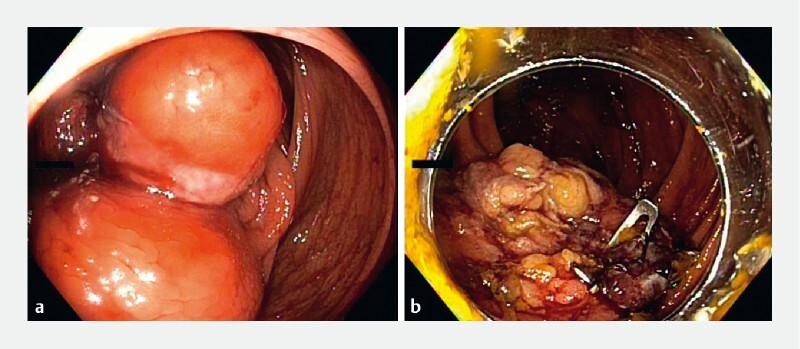
Endoscopic images. **a** The lipoma on index colonoscopy. **b** The colonic lumen after debulking of the lipoma.

**Video 1** Endoscopic debulking of a large colonic lipoma causing recurrent intussusception using endoscopic mucosotomy technique.


Endoscopy_UCTN_Code_TTT_1AQ_2AD

## Notes

Endoscopy E-Videos

https://eref.thieme.de/e-videos
*E-Videos* is an open access online section of the journal *Endoscopy* , reporting on interesting cases and new techniques in gastroenterological endoscopy. All papers include a high-quality video and are published with a Creative Commons CC-BY license. Endoscopy E-Videos qualify for HINARI discounts and waivers and eligibility is automatically checked during the submission process. We grant 100% waivers to articles whose corresponding authors are based in Group A countries and 50% waivers to those who are based in Group B countries as classified by Research4Life (see: https://www.research4life.org/access/eligibility/ ). This section has its own submission website at https://mc.manuscriptcentral.com/e-videos
